# Nomograms for intraoperative prediction of lymph node metastasis in clinical stage IA lung adenocarcinoma

**DOI:** 10.1002/cam4.6115

**Published:** 2023-05-22

**Authors:** Feng Li, Suokai Zhai, Li Fu, Lin Yang, Yousheng Mao

**Affiliations:** ^1^ Department of Thoracic Surgery National Cancer Center/National Clinical Research Center for Cancer/Cancer Hospital, Chinese Academy of Medical Sciences and Peking Union Medical College Beijing China; ^2^ Department of Thoracic Surgery The Cancer Hospital of the University of Chinese Academy of Sciences (Zhejiang Cancer Hospital), Institute of Basic Medicine and Cancer (IBMC), Chinese Academy of Sciences Hangzhou China; ^3^ Department of Cardiothoracic Surgery Zibo First Hospital, Weifang Medical University Zibo Shandong Province China; ^4^ Department of Pathology National Cancer Center/National Clinical Research Center for Cancer/Cancer Hospital, Chinese Academy of Medical Sciences and Peking Union Medical College Beijing China

**Keywords:** lung adenocarcinoma, lymph node metastasis, nomogram, prediction

## Abstract

**Background:**

Accurate prediction of lymph node metastasis (LNM) is critical for selecting optimal surgical procedures in early‐stage lung adenocarcinoma (LUAD). This study aimed to develop nomograms for intraoperative prediction of LNM in clinical stage IA LUAD.

**Methods:**

A total of 1227 patients with clinical stage IA LUADs on computed tomography (CT) were enrolled to construct and validate nomograms for predicting LNM (LNM nomogram) and mediastinal LNM (LNM‐N2 nomogram). Recurrence‐free survival (RFS) and overall survival (OS) were compared between limited mediastinal lymphadenectomy (LML) and systematic mediastinal lymphadenectomy (SML) in the high‐ and low‐risk groups for LNM‐N2, respectively.

**Results:**

Three variables were incorporated into the LNM nomogram and the LNM‐N2 nomogram, including preoperative serum carcinoembryonic antigen (CEA) level, CT appearance, and tumor size. The LNM nomogram showed good discriminatory performance, with C‐indexes of 0.879 (95% CI, 0.847–0.911) and 0.880 (95% CI, 0.834–0.926) in the development and validation cohorts, respectively. The C‐indexes of the LNM‐N2 nomogram were 0.812 (95% CI, 0.766–0.858) and 0.822 (95% CI, 0.762–0.882) in the development and validation cohorts, respectively. LML and SML had similar survival outcomes among patients with low risk of LNM‐N2 (5‐year RFS, 88.1% vs. 89.5%, P*p* = 0.790; 5‐year OS, 96.0% vs. 93.0%, *p* = 0.370). However, for patients with high risk of LNM‐N2, LML was associated with worse survival (5‐year RFS, 64.0% vs. 77.4%, *p* = 0.036; 5‐year OS, 66.0% vs. 85.9%, *p* = 0.038).

**Conclusions:**

We developed and validated nomograms to predict LNM and LNM‐N2 intraoperatively in patients with clinical stage IA LUAD on CT. These nomograms may help surgeons to select optimal surgical procedures.

## 1 INTRODUCTION

Lobectomy with systematic lymph node (LN) dissection is the standard surgical procedure for patients with non‐small‐cell lung cancer (NSCLC).^1^ However, with the advent of low‐dose computed tomography (CT) screening, the detection rate of small‐sized NSCLCs is increasing.^2^ Small NSCLCs are less aggressive in nature and have a lower probability of LN metastases, suggesting potential possibility for sublobar resection and limited mediastinal lymphadenectomy (LML).^3^, ^4^, ^5^ Previous studies have shown that both the incidence of early‐stage NSCLC and the use of sublobar resection and LML are increasing.^6^, ^7^, ^8^

Sublobar resection has the advantages of preserving pulmonary function, reducing perioperative morbidity, and improving patients' postoperative quality of life, especially for patients with multiple comorbidities and poor cardiopulmonary function.^9^ LML, compared with systematic mediastinal lymphadenectomy (SML), is associated with reduced damage of adjacent mediastinal structures, lower incidence of complications and shortened hospitalization.^4^, ^10^ However, inadequate mediastinal nodal dissection may underestimate the nodal stage of some N2‐positive patients, thus depriving these patients of the opportunity to receive adjuvant therapy.^11^, ^12^ Besides, when applying sublobar resection, it is essential to confirm that there is no LN metastasis.^13^, ^14^ Therefore, accurate prediction of the LN status is critical for selecting the optimal treatment in clinical early‐stage NSCLC.

Nevertheless, even with current diagnostic technologies, accurately predicting the pathological LN status preoperatively is still difficult. Conventionally, for small‐sized NSCLCs, surgeons determine nodal status using non‐invasive methods, including CT and positron emission tomography (PET) scan. However, even in patients with clinical stage IA lung adenocarcinoma (LUAD) identified by both CT and PET, the incidence of lymph node metastasis (LNM) is still high.^15^, ^16^, ^17^ Thus, sublobar resection and LML should not be standard procedures, but selective strategies for early‐stage NSCLC. The key to an optimal selective strategy is establishing a reliable and clinically applicable approach that can accurately predict LNM. Currently, there is no such method.

In the present work, we attempted to identify factors associated with LNM in a large cohort of patients with clinical stage IA LUADs and develop prediction models that can determine the risk of LNM intraoperatively. Moreover, we evaluated the clinical applicability of the developed models to figure out if they can help surgeons select optimal surgical procedures (sublobar resection vs. lobectomy and LML vs. SML).

## 2 MATERIALS AND METHODS

### 2.1 Study Cohort

**FIGURE 1:**
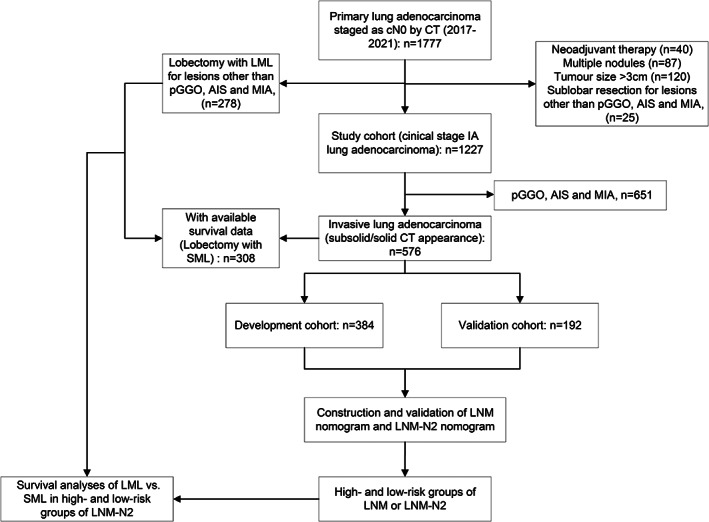
Flow chart of the study. CT, computed tomography; pGGO, pure ground‐glass opacity; AIS, adenocarcinoma in situ; MIA, minimally invasive adenocarcinoma; LML, limited mediastinal lymphadenectomy; SML, systematic mediastinal lymphadenectomy; LNM, lymph node metastasis; LNM‐N2, mediastinal LNM.

This study included a cohort of patients with LUAD who underwent surgical resection at our center between January 2017 and October 2021. All patients underwent contrast‐enhanced chest CT scan, brain magnetic resonance imaging or CT scan, abdominal CT scan or ultrasonography, and bone scan before surgery. Some patients underwent PET scan. Because PET is expensive in China, patients underwent this examination only if they could afford it (*n* = 196). Patients were included if they (1) were clinical stage IA: had disease 3 cm and no enlarged LNs (the shortest axis of LNs >1 cm) or distant metastasis based on preoperative examination, and (2) underwent lobectomy with SML except for pure ground‐glass opacity (pGGO) lesions, adenocarcinoma in situ (AIS) or minimally invasive adenocarcinoma (MIA). The exclusion criteria were as follows: (1) patients with tumors >3 cm or enlarged LNs on CT scan; (2) patients who received neoadjuvant treatments before surgery; (3) patients with multiple lung nodules. Finally, 1227 patients were eligible for evaluation. Besides, as this study specifically excluded patients who did not undergo SML, to evaluate the survival impact of the extent of lymphadenectomy, 278 patients who underwent lobectomy with LML from the same time frame of the study were also included. Survival differences (LML vs. SML) was tested between this group of patients and 308 patients with available survival data from the primary cohort. The flow chart of the study is presented in Figure 1. This retrospective cohort study was approved by the institutional review board of our center (NCC2014ST‐07). The requirement to obtain informed consent was waived due to the retrospective design of the study. We have registered this study in Chinese Clinical Trial Registry (Registration number: ChiCTR2200061299).

### 2.2 Definitions of SML and LML

SML is defined as en‐block removal of the fatty tissue and lymph nodes within the anatomic mediastinal stations (2R, 4R, 7, 8, 9 for right‐side tumors and 4L, 5, 6, 7, 8, 9 for left‐side tumors) according to the recommendations of the European Society of Thoracic Surgeons.^18^ The hilar, lobar, and interlobar LNs (stations 10–14) were removed as a part of lung resection. LML includes selective mediastinal LN dissection, and incomplete mediastinal LN sampling or dissection. It was performed after comprehensive evaluation by the surgeons. Possible candidates for LML included old patients with poor performance status, patients with complex comorbidities or reduced cardiopulmonary reserve, and patients for whom the surgeons determined that SML posed too great a risk during surgery. Besides, LML was performed conventionally for small NSCLCs considered to be clinically node‐negative.

### 2.3 Histologic Evaluation

All paraffin‐embedded tumor specimens were reviewed by two pathologists. Any disagreement between the pathologists was resolved using a multiheaded microscope and reached a consensus ultimately. Tumors were classified, in accordance with the 2015 World Health Organization (WHO) classification of lung tumors, as AIS, MIA, and invasive adenocarcinoma (IAC) which was further subdivided into six subtypes: lepidic, acinar, papillary, micropapillary, and solid predominant subtypes, as well as invasive mucinous adenocarcinoma. The percentage of each histological pattern was semiquantitatively recorded in 5% increments. If 5% or greater of a specific histological pattern was found, it was considered as having the presence of this pattern. Tumor spread through air spaces (STAS), visceral pleural invasion (VPI), and lymphovascular invasion (LVI) were also evaluated based on the paraffin‐embedded specimens.

### 2.4 Evaluation of LNM

Pathological records were retrospectively reviewed, and the presence and location of LNMs were identified for all patients in the primary cohort. LNMs were classified as intrapulmonary LNMs (only stations 11–14), hilar LNMs (station 10, with or without intrapulmonary LNMs), and mediastinal LNMs (stations 2–9, with or without intrapulmonary and hilar LNMs) according to the location of LNMs.

### 2.5 Patient Follow‐up

Postoperative follow‐up was carried out every 3 months within the first 2 years and every 6 months from the third year. Recurrence‐free survival (RFS) was defined as the interval from the date of surgery until the first recurrence or last follow‐up. Overall survival (OS) was defined as the interval from the date of surgery until death or last follow‐up.

### 2.6 Statistical analysis

T‐test and Pearson chi‐squared test were used to evaluate differences between continuous and categorical variables, respectively. Two nomograms were constructed in this study: one for predicting LNM and another for mediastinal LNM (LNM‐N2). To evaluate the performance of the nomograms, the primary cohort was randomly split into a development cohort (consisting of two thirds of the patients) and a validation cohort (consisting of the remaining one third). Univariate and multivariate logistic regression analyses were performed to identify variables related to LNM and LNM‐N2 in the development cohort. As pathological tumor size was unavailable before resection, the maximum tumor size determined by CT (including the ground‐glass opacity components) was used as a surrogate. Nomograms were developed on the basis of the final logistic models.

The predictive ability of the nomograms was evaluated by Harrell's concordance index (C‐index) and calibration curves. Decision curves were used to evaluate the value of the nomograms in clinical practice. For the prediction nomograms to be clinically applicable, cutoff points for the probability of LNM and LNM‐N2 were selected. We used receiver operating characteristic (ROC) curves and Youden Index to determine the optimal cutoff points. The Kaplan–Meier method was used to estimate RFS and OS, and survival differences between groups were evaluated using the log‐rank test. Multivariate Cox regression analyses were used to evaluate the independent effect of extent of lymphadenectomy (LML vs. SML) on survival. All statistical tests were two‐sided and *p* < 0.05 was considered statistically significant. The statistical analyses were conducted using STATA 16.0 (STATA Corp) and R 4.0.2 (R Foundation for Statistical Computing).

## 3 RESULTS

### 3.1 Patient characteristics

Among 1227 patients from the primary cohort, 818 were randomly assigned to the development cohort and 409 to the validation cohort. Baseline clinicopathological characteristics of the development and validation cohorts are summarized in Table 1. 212 (17.3%) patients had LNMs. Of the 212 patients with LNMs, 35.4% had only intrapulmonary LNMs, 4.7% hilar LNMs, and 59.9% mediastinal LNMs. Only one patient with pGGO had LNMs and no patients with AIS and MIA had LNM.

**TABLE 1:** Clinicopathological variables of the development and validation cohorts.

	Development cohort						Validation cohort					
Variables	LNM (+) (*n* = 140)	LNM (−) (*n* = 678)	*p* value	LNM‐N2 (+) (*n* = 82)	LNM‐N2 (−) (*n* = 736)	*p* value	LNM (+) (*n* = 72)	LNM (−) (*n* = 337)	*p* value	LNM‐N2 (+) (*n* = 45)	LNM‐N2 (−) (*n* = 364)	*p* value
Age, years												
≦60	82 (58.6)	422 (62.2)	0.416	52 (63.4)	452 (61.4)	0.724	39 (54.2)	225 (66.8)	0.043	26 (57.8)	238 (65.4)	0.314
>60	58 (41.4)	256 (37.8)		30 (36.6)	284 (38.6)		33 (45.8)	112 (33.2)		19 (42.2)	126 (34.6)	
Sex												
Female	82 (58.6)	476 (70.2)	0.007	44 (53.7)	514 (69.8)	0.003	42 (58.3)	223 (66.2)	0.206	31 (68.9)	234 (64.3)	0.542
Male	58 (41.4)	202 (29.8)		38 (46.3)	222 (30.2)		30 (41.7)	114 (33.8)		14 (31.1)	130 (35.7)	
Smoking												
Never	101 (72.1)	571 (84.2)	0.001	57 (69.5)	615 (83.6)	0.002	51 (70.8)	274 (81.3)	0.046	33 (73.3)	292 (80.2)	0.281
Former/Current	39 (27.9)	107 (15.8)		25 (30.5)	121 (16.4)		21 (29.2)	63 (18.7)		12 (26.7)	72 (19.8)	
CEA, ng/mL												
≧5	68 (48.6)	36 (5.3)	<0.001	42 (51.2)	62 (8.4)	<0.001	45 (62.5)	15 (4.5)	<0.001	18 (40.0)	24 (6.6)	<0.001
<5	72 (51.4)	642 (94.7)		40 (48.8)	674 (91.6)		27 (37.5)	322 (95.5)		27 (60.0)	340 (93.4)	
Tumor location												
Right upper lobe	41 (29.3)	233 (34.5)	0.400	27 (32.9)	247 (33.7)	0.197	24 (33.3)	107 (31.7)	0.599	14 (31.1)	117 (32.1)	0.734
Right middle lobe	13 (9.3)	61 (9.0)		7 (8.5)	67 (9.1)		7 (9.7)	19 (5.6)		2 (4.4)	24 (6.6)	
Right lower lobe	28 (20.0)	101 (14.9)		20 (24.4)	109 (14.8)		10 (13.9)	67 (19.9)		7 (15.6)	70 (19.2)	
Left upper lobe	39 (27.9)	167 (24.7)		19 (23.2)	187 (25.5)		22 (30.6)	104 (30.9)		14 (31.1)	112 (30.8)	
Left lower lobe	19 (13.6)	114 (16.9)		9 (11.0)	124 (16.9)		9 (12.5)	40 (11.9)		8 (17.8)	41 (11.3)	
CT tumor size, cm mean (SD)	2.2 (0.6)	1.4 (0.6)	<0.001	2.2 (0.6)	1.5 (0.7)	<0.001	2.2 (0.6)	1.4 (0.6)	<0.001	2.2 (0.6)	1.5 (0.6)	<0.001
CT tumor size, cm												
≦1	4 (2.9)	230 (33.9)	<0.001	3 (3.7)	231 (31.4)	<0.001	2 (2.8)	108 (32.0)	<0.001	2 (4.4)	108 (29.7)	<0.001
1–2	51 (36.4)	333 (49.1)		34 (41.4)	350 (47.5)		20 (27.8)	170 (50.5)		13 (28.9)	177 (48.6)	
2–3	85 (60.7)	115 (17.0)		45 (54.9)	155 (21.1)		50 (69.4)	59 (17.5)		30 (66.7)	79 (21.7)	
CT appearance												
pGGO	1 (0.7)	336 (49.5)	<0.001	0 (0.0)	337 (45.8)	<0.001	0 (0.0)	167 (49.6)	<0.001	0 (0.0)	167 (45.9)	<0.001
Part‐solid	4 (2.9)	210 (31.0)		2 (2.4)	212 (28.8)		3 (4.2)	115 (34.1)		1 (2.2)	117 (32.1)	
Pure‐solid	135 (96.4)	132 (19.5)		80 (97.6)	187 (25.4)		69 (95.8)	55 (16.3)		44 (97.8)	80 (22.0)	
Consolidation/Tumor ratio												
≧50%	137 (97.9)	209 (30.8)	<0.001	81 (98.8)	265 (36.0)	<0.001	72 (100.0)	96 (28.5)	<0.001	45 (100.0)	123 (33.8)	<0.001
<50%	3 (2.1)	469 (69.2)		1 (1.2)	471 (64.0)		0 (0.0)	241 (71.5)		0 (0.0)	241 (66.2)	
Invasion status												
AIS	0 (0.0)	100 (14.7)	<0.001	0 (0.0)	100 (13.6)	<0.001	0 (0.0)	53 (15.7)	<0.001	0 (0.0)	53 (14.6)	<0.001
MIA	0 (0.0)	240 (35.4)		0 (0.0)	240 (32.6)		0 (0.0)	114 (33.8)		0 (0.0)	114 (31.3)	
IAC	140 (100.0)	338 (49.9)		82 (100.0)	396 (53.8)		72 (100.0)	170 (50.5)		45 (100.0)	197 (54.1)	
Pathologic tumor size, cm mean (SD)	2.3 (0.7)	1.2 (0.7)	<0.001	2.2 (0.7)	1.3 (0.7)	<0.001	2.3 (0.8)	1.3 (0.6)	<0.001	2.2 (0.8)	1.3 (0.7)	<0.001
Visceral pleural invasion												
Absent	73 (52.1)	649 (95.7)	<0.001	42 (51.2)	680 (92.4)	<0.001	35 (48.6)	325 (96.4)	<0.001	19 (42.2)	341 (93.7)	<0.001
Present	67 (47.9)	29 (4.3)		40 (48.8)	56 (7.6)		37 (51.4)	12 (3.6)		26 (57.8)	23 (6.3)	
Lymphovascular invasion												
Absent	82 (58.6)	666 (98.2)	<0.001	43 (52.4)	705 (95.8)	<0.001	34 (47.2)	329 (97.6)	<0.001	20 (44.4)	343 (94.2)	<0.001
Present	58 (41.4)	12 (1.8)		39 (47.6)	31 (4.2)		38 (52.8)	8 (2.4)		25 (55.6)	21 (5.8)	
Predominant subtype												
Lepidic	3 (2.2)	118 (34.9)	<0.001	2 (2.5)	119 (30.1)	<0.001	2 (2.8)	67 (39.4)	<0.001	1 (2.2)	68 (34.5)	0.001
Acinar	87 (62.1)	146 (43.2)		50 (61.0)	183 (46.2)		41 (56.9)	80 (47.1)		30 (66.7)	91 (46.2)	
Papillary	21 (15.0)	47 (13.9)		12 (14.6)	56 (14.1)		12 (16.7)	15 (8.8)		7 (15.6)	20 (10.2)	
Micropapillary	9 (6.4)	4 (1.2)		5 (6.1)	8 (2.0)		7 (9.7)	1 (0.6)		2 (4.4)	6 (3.0)	
Solid	18 (12.9)	7 (2.1)		12 (14.6)	13 (3.3)		10 (13.9)	3 (1.8)		5 (11.1)	8 (4.1)	
IMA	2 (1.4)	16 (4.7)		1 (1.2)	17 (4.3)		0 (0.0)	4 (2.3)		0 (0.0)	4 (2.0)	
STAS												
Absent	14 (10.0)	634 (93.5)	<0.001	11 (13.4)	637 (86.6)	<0.001	10 (13.9)	320 (95.0)	<0.001	7 (15.6)	323 (88.7)	<0.001
Present	126 (90.0)	44 (6.5)		71 (86.6)	99 (13.4)		62 (86.1)	17 (5.0)		38 (84.4)	41 (11.3)	
Micropapillary pattern												
Absent	40 (29.0)	260 (80.7)	<0.001	25 (30.9)	275 (72.6)	<0.001	15 (20.8)	140 (84.3)	<0.001	10 (22.2)	145 (75.1)	<0.001
Present	98 (71.0)	62 (19.3)		56 (69.1)	104 (27.4)		57 (79.2)	26 (15.7)		35 (77.8)	48 (24.9)	
Solid pattern												
Absent	74 (53.6)	288 (89.4)	<0.001	44 (54.3)	318 (83.9)	<0.001	43 (59.7)	150 (90.4)	<0.001	26 (57.8)	167 (86.5)	<0.001
Present	64 (46.4)	34 (10.6)		37 (45.7)	61 (16.1)		29 (40.3)	16 (9.6)		19 (42.2)	26 (13.5)	
Lepidic pattern												
Absent	90 (65.2)	79 (24.5)	<0.001	48 (59.3)	121 (31.9)	<0.001	47 (65.3)	26 (15.7)	<0.001	28 (62.2)	45 (23.3)	<0.001
Present	48 (34.8)	243 (75.5)		33 (40.7)	258 (68.1)		25 (34.7)	140 (84.3)		17 (37.8)	148 (76.7)	
Mutation status (*n* = 744)												
EGFR	73 (59.3)	204 (61.6)	0.538	49 (64.5)	228 (60.3)	0.442	43 (60.5)	102 (65.4)	0.465	30 (68.2)	115 (62.8)	0.192
KRAS	12 (9.8)	40 (12.1)		6 (7.9)	46 (12.2)		7 (9.9)	16 (10.3)		1 (2.3)	22 (12.0)	
BRAF	1 (0.8)	6 (1.8)		0 (0.0)	7 (1.8)		0 (0.0)	3 (1.9)		0 (0.0)	3 (1.7)	
Wild type	37 (30.1)	81 (24.5)		21 (27.6)	97 (25.7)		21 (29.6)	35 (22.4)		13 (29.5)	43 (23.5)	
LNM location (*n* = 212)												
Intrapulmonary	52 (37.1)						23 (31.9)					
Hilar	6 (4.3)						4 (5.6)					
Mediastinal	82 (58.6)						45 (62.5)					

Abbreviations: AIS, adenocarcinoma in situ; CEA, serum carcinoembryonic antigen; CT, computed tomography; IAC, invasive adenocarcinoma; IMA, invasive mucinous adenocarcinoma; LNM, lymph node metastasis; LNM‐N2, mediastinal LNM; MIA, minimally invasive adenocarcinoma; pGGO, pure ground‐glass opacity; SD, standard deviation; STAS, tumor spread through air spaces.

The presence of LNM and LNM‐N2 was associated with smoking, elevated preoperative serum carcinoembryonic antigen (CEA) level, larger tumor size, solid CT appearance, higher consolidation/tumor ratio, invasive adenocarcinoma, presence of STAS, LVI, and VPI, presence of micropapillary and solid patterns and absence of lepidic pattern (Table 1). Patients with micropapillary/solid predominant subtypes had significantly higher risks of LNM and LNM‐N2 while patients with lepidic predominant subtype had significantly lower risks. No significant relationship with LNM and LNM‐N2 was found for age, tumor location, and mutation status.

### 3.2 Development and validation of the nomogram for predicting LNM

Patients with pGGO, AIS, and MIA were excluded for the development of the nomograms because almost none of them had LNM (Figure 1). Ultimately, 576 patients were retained: 384 in the development cohort and 192 in the validation cohort. Only variables available preoperatively were selected for the nomograms. Multivariable analysis (Table 2) revealed that elevated serum CEA level [odds ratio (OR), 6.06; 95% confidence interval (CI), 3.11–11.83; *p* < 0.001], pure solid appearance (OR, 24.21; 95% CI, 5.47–107.21; *p* < 0.001), and larger CT tumor size (OR, 2.84; 95% CI, 1.79–4.51; *p* < 0.001) were associated with LNM.

**TABLE 2:** Univariable and multivariable logistic regression analyses for predicting lymph node metastasis.

	LNM				LNM‐N2			
	Univariable analysis		Multivariable analysis		Univariable analysis		Multivariable analysis	
	OR (95% CI)	*p* value	OR (95% CI)	*p* value	OR (95% CI)	*p* value	OR (95% CI)	*p* value
Age, years	0.99 (0.97–1.01)	0.415			0.98 (0.96–1.01)	0.154		
Sex								
Female	Reference				Reference			
Male	1.10 (0.72–1.69)	0.655			1.34 (0.82–2.19)	0.250		
Smoking history								
Never	Reference				Reference			
Former/Current	1.40 (0.87–2.27)	0.167			1.48 (0.86–2.57)	0.159		
CEA, ng/mL								
<5	Reference		Reference		Reference		Reference	
≧5	10.25 (5.81–18.06)	<0.001	6.06 (3.11–11.83)	<0.001	6.03 (3.52–10.36)	<0.001	3.41 (1.91–6.08)	<0.001
Tumor location								
Right upper lobe	Reference				Reference			
Right middle lobe	1.23 (0.56–2.68)	0.610			0.89 (0.35–2.26)	0.804		
Right lower lobe	1.42 (0.77–2.61)	0.263			1.45 (0.74–2.84)	0.278		
Left upper lobe	1.46 (0.83–2.56)	0.186			0.93 (0.48–1.79)	0.818		
Left lower lobe	0.79 (0.41–1.54)	0.496			0.50 (0.21–1.17)	0.111		
CT appearance								
Part solid	Reference		Reference		Reference		Reference	
Pure solid	39.42 (14.14–109.96)	<0.001	24.21 (5.47–107.21)	<0.001	32.17 (7.76–133.28)	<0.001	17.49 (2.34–130.48)	0.005
Consolidation/Tumor ratio								
<50%	Reference		Reference		Reference		Reference	
≧50%	32.94 (7.95–136.42)	<0.001	1.72 (0.22–13.16)	0.603	29.68 (4.06–216.80)	0.001	1.60 (0.10–26.34)	0.744
CT tumor size, cm	2.69 (1.87–3.86)	<0.001	2.84 (1.79–4.51)	<0.001	1.91 (1.27–2.85)	0.002	1.55 (0.98–2.47)	0.061

Abbreviations: CI, confidence interval; CEA, serum carcinoembryonic antigen; CT, computed tomography; LNM, lymph node metastasis; LNM‐N2, mediastinal LNM; OR, odds ratio; STAS, tumor spread through air spaces.

**FIGURE 2:**
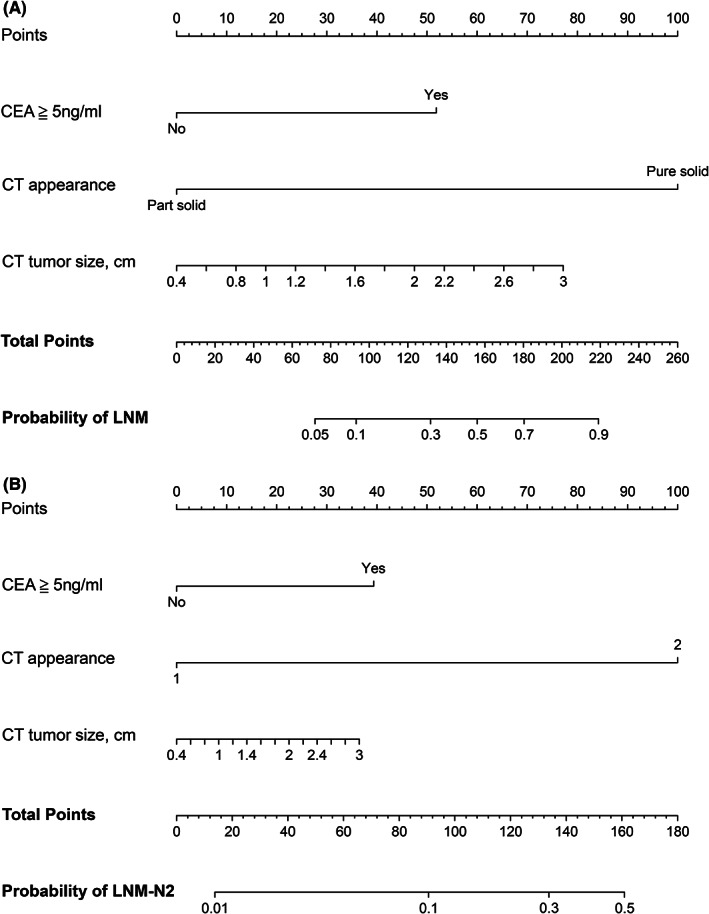
Nomograms for intraoperative prediction of LNM (A) and LNM‐N2 (B) in clinical stage IA lung adenocarcinoma. LNM, lymph node metastasis; LNM‐N2, mediastinal LNM; CEA, serum carcinoembryonic antigen.

**FIGURE 3:**
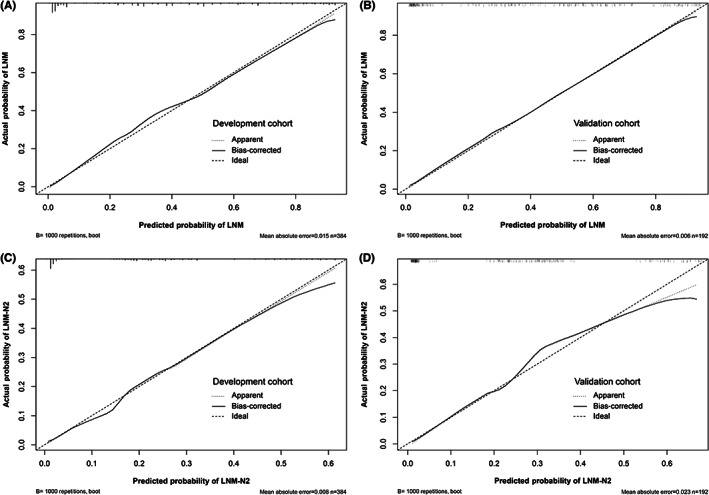
Calibration curves of the nomograms for predicting LNM (A, B) and LNM‐N2 (C, D) in the development and validation cohorts. LNM, lymph node metastasis; LNM‐N2, mediastinal LNM.

The nomogram incorporating the above three variables for predicting LNM (LNM nomogram) was constructed (Figure 2A). For convenience of clinical use, detailed point assignments for each variable in the nomogram are presented in Table 3. The nomogram could assign the probability of LNM using the sum of the points identified for each variable. The mark for total points aligned with the bottom scale indicated the risk of LNM. The nomogram showed good discriminatory performance, with C‐indexes of 0.879 (95% CI, 0.847–0.911) in the development cohort and 0.880 (95% CI, 0.834–0.926) in the validation cohort. The calibration curves (Figures 3A, B) showed high concordance between predicted and actual probabilities.

**TABLE 3:** Detailed points of each predictor in the nomograms.

Variables	Points for LNM nomogram	Points for LNM‐N2 nomogram
CT appearance		
Part solid	0	0
Pure solid	100	100
CEA, ng/mL		
<5	0	0
≧5	52	39
CT tumor size, cm	30*(Size‐0.4)	14*(Size‐0.4)
Cutoff points	136	116.8

Abbreviations: CEA, serum carcinoembryonic antigen; CT, computed tomography; LNM, lymph node metastasis; LNM‐N2, mediastinal LNM; STAS, tumor spread through air spaces.

### 3.3 Development and validation of the nomogram for predicting LNM‐N2

As presented in Table 2, elevated serum CEA level (OR, 3.41; 95% CI, 1.91–6.08; *p* < 0.001) and pure solid appearance (OR, 17.49; 95% CI, 2.34–130.48; *p* = 0.005) were significant predictors of LNM‐N2 in the multivariate analysis. Larger CT tumor size was also associated with LNM‐N2, with a marginal statistical significance (OR, 1.55; 95% CI, 0.98–2.47; *p* = 0.061). Given its clinical relevance, tumor size was also incorporated into the nomogram.

The nomogram for predicting LNM‐N2 (LNM‐N2 nomogram) was constructed based on the above three variables (Figure 2B). Detailed points of each variable in the nomogram are shown in Table 3. The C‐indexes of the LNM‐N2 nomogram were 0.812 (95% CI, 0.766–0.858) and 0.822 (95% CI, 0.762–0.882) in the development and validation cohorts, respectively. The calibration curves (Figures 3C, D) showed good agreement between predicted and actual probabilities.

### 3.4 Clinical value of the nomograms

After obtaining risk points for the entire cohort population from the nomograms, risk stratification was conducted using the maximum Youden Index. The optimal cutoff point for LNM nomogram was 136 and the optimal cutoff point for LNM‐N2 nomogram was 116.8 (Figure S1, Table 3). Accordingly, the optimal cutoff probability of LNM was 0.33 and the optimal cutoff probability of LNM‐N2 was 0.20 (Figure 2). Decision curve analyses suggested that when the threshold probabilities of LNM and LNM‐N2 were 0.33 and 0.20, respectively, the net benefits of the nomograms were significantly higher compared with the treat‐all or treat‐none schemes (Figure S2). Using 136 as a cutoff point to identify LNM, the LNM nomogram had an area under ROC (AUC) of 0.877 in the entire cohort. AUC of the LNM‐N2 nomogram was 0.813 when the cutoff point was 116.8 (Figure S1).

Then the patients were classified into low‐ and high‐risk groups, respectively, according to the optimal cutoff point (LNM nomogram: 136). The low‐risk group had noticeably decreased possibilities of LNM (Figure S3). The probability of intrapulmonary LNM decreased from 22.88% in the high‐risk group for LNM to 3.61% in the low‐risk group for LNM and the probability of mediastinal LNM decreased from 39.11% to 6.56% (Figure S3a).

**FIGURE 4:**
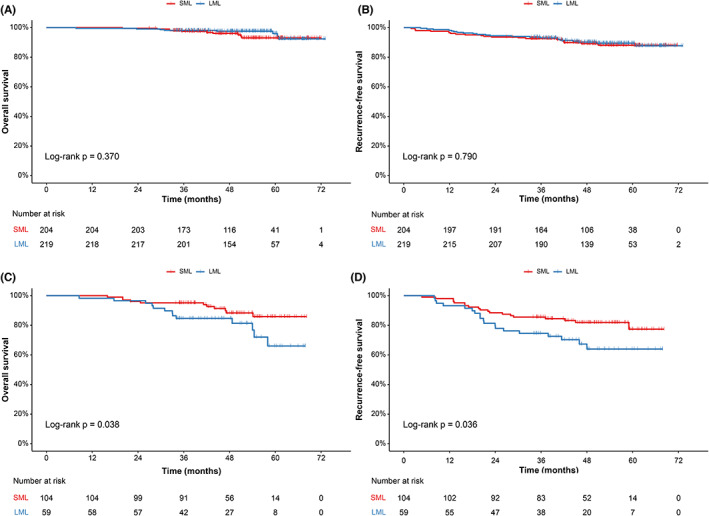
Overall survival and recurrence‐free survival of SML and LML in the low‐risk group for LNM‐N2 (A, B) and high‐risk group for LNM‐N2 (C, D). SML, systematic mediastinal lymphadenectomy; LML, limited mediastinal lymphadenectomy; LNM‐N2, mediastinal lymph node metastasis.

An additional analysis was performed to evaluate the clinical performance of the risk stratification of mediastinal LNM (according to LNM‐N2 nomogram) for selecting extent of lymphadenectomy (LML vs. SML). As shown in Figure 4A, B, there was no difference in RFS (5‐year survival, 88.1% vs. 89.5%, *p* = 0.790) and OS (5‐year survival, 96.0% vs. 93.0%, *p* = 0.370) between LML and SML in the low‐risk group for LNM‐N2. However, in the high‐risk group, LML was associated with worse RFS (5‐year survival, 64.0% vs. 77.4%, *p* = 0.036) and OS (5‐year survival, 66.0% vs. 85.9%, *p* = 0.038) than SML (Figure 4C, D). After multivariate adjustment, LML remained as an independent predictor of worse RFS (HR: 4.00, 95% CI: 1.82–8.75, *p* = 0.001) and OS (HR: 3.30, 95% CI: 1.44–7.57, *p* = 0.005) in the high‐risk group for LNM‐N2, but not in the low‐risk group for LNM‐N2 (Table S1). The clinicopathological characteristics of the patients who underwent SML or LML with available survival data are shown in Table S2.

## 4 DISCUSSION

With the rising detection rate of small lung nodules, the emerging challenge for thoracic surgeons now is how to use function‐preserving surgical methods, such as sublobar resection and LML, without compromising the oncological outcomes. On the one hand, when applying sublobar resection, prediction of the pathological N0 status is crucial. Normally, all hilar and lobe‐specific mediastinal LNs need to be examined intraoperatively to confirm N0.^13^, ^14^ However, intraoperative examination of many LNs is not realistic. On the other hand, LML may understage the tumor, making patients with LNM miss the opportunity of adjuvant therapy and further resulting in poor prognosis.^11^, ^12^ Therefore, accurate nodal staging is of paramount importance when considering these function‐preserving surgical methods.

LNMs are common even in patients with clinical N0 small NSCLC.^15^, ^16^, ^17^, ^19^, ^20^ Studies have shown that nodal staging by CT or PET scan may understage 10–20% of patients.^17^, ^19^, ^20^ Consistently, in this study, 17.3% of patients with clinical stage IA LUADs based on CT scan were upstaged after SML (6.9% with pN1 and 10.4% with pN2). Besides, in consistent with previous studies,^17^, ^21^ more than one‐third of the LNMs were found to be intrapulmonary LNMs. These LNMs would likely not have been found by wedge resection and thus these patients might be understaged and undertreated. Therefore, accurately predicting the risk of LNM is crucial for selecting candidates for sublobar resection and LML.

By using a large clinical cohort, we identified risk factors associated with the occurrence of LNM and LNM‐N2. Furthermore, we developed prediction nomograms which combined variables available preoperatively to estimate the probability of LNM and LNM‐N2. The prediction nomograms were successfully and independently validated and had good accuracy and calibration in both the development cohort and the validation cohort. They will help surgeons to identify not only patients who could undergo sublobar resection and LML, but also those who are at high risk of unforeseen LNM and for whom lobectomy and SML should be performed.

The predictors in our nomograms have also been identified in other studies. As previous studies demonstrated,^22^, ^23^ solid component was significantly associated with LNM and the probability of LNM increased markedly as the solid component increased. Pure‐solid nodules had a significantly higher rate of LNM than part‐solid nodules. No patients with pGGO had LNM except one had intrapulmonary LNMs. The 5‐year postoperative survival of patients with pGGO was reported to approach 100% after sublobar resection.^24^ Therefore, sublobar resection and omission of LN dissection may be feasible for these patients. The role of CEA as a predictor of LNM has been widely studied.^25^, ^26^ Elevated preoperative serum CEA level was associated with pathological nodal upstaging. Tumor size is a well‐known predictor of LNM.^22^, ^27^ As the tumor size increased, the risk of LNM raised rapidly. Tumor size <1 cm used to be suggested as an indicator of pN0 status.^28^, ^29^ However, in both our study and studies by others,^16^, ^27^ LNMs were seen in sub‐centimeter tumors.

Histological features are being increasingly recognized as strong predictors of LNM in LUAD. No patients with AIS and MIA had LNM in this study and it is recommended that these patients undergo function‐preserving surgery given the excellent prognosis after sublobar resection without mediastinal lymphadenectomy.^30^ However, adenocarcinomas with certain histological features, such as STAS, micropapillary, and solid patterns, are more aggressive and associated with high risk of LNM.^17^, ^31^, ^32^ In this study, the presence of micropapillary/solid pattern and STAS was significantly associated with LNM, further emphasizing the importance of histological features in predicting LNM. Identification of these histological features would be helpful for guiding surgical strategy (sublobar resection vs. lobectomy and LML vs. SML), in addition to preoperative findings. Frozen section may be a feasible option. We have previously reported that intraoperative frozen section is reliable for identifying the invasion status of LUAD, with high diagnostic accuracy for differentiating AIS/MIA from IAC.^33^ However, for micropapillary/solid pattern and STAS, current evidence does not warrant frozen section evaluation for the presence of these features due to the unsatisfactory sensitivity.^34^, ^35^ Therefore, in this study, considering the clinical utility, we did not incorporate the presence of these features into the nomograms.

To evaluate the clinical applicability of the nomograms, we stratified the entire cohort into low‐ and high‐risk groups, respectively, according to the cutoff points determined by the nomograms (LNM nomogram: 136; LNM‐N2 nomogram: 116.8). In the entire cohort, more than one‐third (35.4%) of the LNMs were found to be intrapulmonary LNMs. These LNMs would likely not have been found by hilar and mediastinal sampling during sublobar resection, especially wedge resection, and thus these patients might be understaged and undertreated. However, using the risk stratification according to the LNM nomogram, the probability of intrapulmonary LNM decreased from 22.88% in the high‐risk group to 3.61% in the low‐risk group. Thus, for patients in the low‐risk group for LNM, sublobar resection, particularly segmentectomy, may be feasible, while for those in the high‐risk group for LNM, lobectomy should still be performed. Furthermore, we evaluated the impact of the risk stratification of mediastinal LNM according to the LNM‐N2 nomogram on procedure‐specific outcomes (SML vs. LML). We found that LML and SML had similar RFS and OS among patients with low risk of LNM‐N2. Therefore, LML may be feasible for these patients. However, for patients with high risk of LNM‐N2, LML was associated with worse survival and SML should still be the standard procedure.

The workflow of treatment planning for patients with clinical stage IA LUADs by CT is illustrated in Figure S4. Briefly, for a patient with clinical stage IA LUAD, the following clinical pathway is recommended: ①(1) if the nodule is presented as pGGO, surgeons can perform sublobar resection without lymph node dissection; ② if the nodule is presented as part‐solid or pure‐solid, intraoperative frozen section should be conducted; ③ if the nodule is classified as atypical adenomatous hyperplasia (AAH) or AIS or MIA by frozen section, surgeons can perform sublobar resection without lymph node dissection; ④ if the nodule is classified as IAC, surgeons should select optimal surgical procedures (sublobar resection vs. lobectomy and LML vs. SML) according to the risk stratification of LNM and LNM‐N2 by the nomograms.

Previous studies have also constructed several models for predicting LNM in NSCLC. Zhang and colleagues developed a 4‐predictor model for N2 disease in T1N0 NSCLC.^36^ Their model was constructed based on both LUAD and squamous‐cell carcinoma (SCC). However, studies have reported that SCC patients have a higher rate of LNM than LUAD patients and that SCC patients are more prone to have N1 disease while LUAD patients have more N2 disease.^27^, ^37^, ^38^, ^39^ Therefore, models for predicting LNM should be constructed separately for LUAD and SCC. Besides, the C‐index (0.726) of their model is unsatisfactory. Gu and colleagues developed a prediction model based on CT texture features in clinical stage IA LUAD.^40^ However, they only included solitary pulmonary nodules and excluded part‐solid nodules, limiting the generalizability of the model. Besides, the clinical applicability of their model is doubtful because the texture features they used are not routinely provided in the CT scan reports. Instead, the variables incorporated in our nomograms can be easily and quickly obtained in routine clinical practice, and do not add burden to the patient. Aokage and colleagues developed a predictive formula for calculating the probability of pathological LNM in clinical stage IA LUAD with a dominant solid part.^41^ The major weakness of their model is that it did not differentiate between the probabilities of pN1 and pN2 due to the small number of patients with each N status. In this study, we evaluated the risk of LNM and LNM‐N2 separately, considering that optimal candidates for sublobar resection and LML may be different. Besides, the diagnostic accuracy of our model was superior to theirs (development cohort: 0.879 vs. 0.804; validation cohort: 0.880 vs. 0.797). More importantly, all the above studies did not provide thresholds or evaluate the clinical utility of their models. Instead, for our nomograms, definite cutoff points were established and the clinical applicability was also evaluated, making them more valuable than their predecessors.

However, there are still some limitations in this study. First, although PET scan is considered to be more accurate regarding non‐invasive assessment of hilar and mediastinal LNs, the preoperative nodal staging in this study was mainly based on CT scan because the high expense of PET scan prohibits its routine application in our country. However, our nomograms can be used in conditions when PET‐CT is not performed and in countries where PET‐CT scan is unaffordable for most patients. Further studies utilizing patients who have received PET‐CT scan to construct nomograms incorporating PET‐CT parameters and other preoperatively available variables should be conducted, which may predict LNM more accurately for small NSCLCs. Second, the number of patients who underwent LML in the high‐risk group for LNM‐N2 was relatively small, which might have affected the results. Third, to reduce the heterogeneity of the study population and improved the accuracy of the nomograms, we excluded patients with multiple lung nodules, which may limit the use of the models for this specific population. Further studies focusing on prediction of LNM in patients with multiple lung cancers should be conducted. Finally, patient selection bias was inevitable due to the retrospective nature of the study. Although multivariate analyses and independent validation could minimize some of these problems and improve the calibration of the prediction nomograms, the generalizability and clinical performance of our nomograms are required to be tested in prospective studies.

In conclusion, we have developed and validated nomograms to predict LNM and LNM‐N2 in patients with clinical stage IA LUAD using preoperatively available variables. Our prediction nomograms may allow surgeons to predict the individualized risk of LNM and LNM‐N2, and select the optimal surgical procedures accordingly.

## AUTHOR CONTRIBUTIONS

**Feng Li:** Conceptualization (equal); data curation (equal); formal analysis (equal); methodology (equal); resources (equal); software (equal); validation (equal); visualization (equal); writing – original draft (equal); writing – review and editing (equal). **Suokai Zhai:** Conceptualization (equal); formal analysis (equal); investigation (equal); software (equal); validation (equal); writing – original draft (equal); writing – review and editing (equal). **Li Fu:** Data curation (equal); formal analysis (supporting); methodology (supporting); software (supporting); validation (supporting); visualization (supporting); writing – original draft (supporting); writing – review and editing (supporting). **Lin Yang:** Data curation (equal); writing – original draft (supporting); writing – review and editing (supporting). **Yousheng Mao:** Conceptualization (lead); project administration (lead); resources (equal); supervision (lead); writing – original draft (equal); writing – review and editing (equal).

## FUNDING INFORMATION

This research did not receive any specific grant from funding agencies in the public, commercial, or not‐for‐profit sectors.

## CONFLICT OF INTEREST STATEMENT

None of the authors have conflicts of interests or disclosures.

## ETHICAL APPROVAL STATEMENT

This retrospective cohort study was approved by the institutional review board of our center (NCC2014ST‐07). The requirement to obtain informed consent was waived due to the retrospective design of the study.

## REGISTRATION NUMBER

This study was registered in Chinese Clinical Trial Registry (Registration number: ChiCTR2200061299).

## DISCLOSURE

The authors declare that they have no known competing financial interests or personal relationships that could have appeared to influence the work reported in this paper.

## Supporting information


**Table S1.** Univariable and multivariable analyses for recurrence‐free and overall survival

**Table S2.** Baseline characteristics of patients undergoing systematic or limited mediastinal lymphadenectomy

**Figure S1:** ROC curves of the nomograms for predicting LNM (A) and LNM‐N2 (B) according to the optimal cutoff points determined by the maximum Youden Index. ROC, receiver operating characteristic; LNM, lymph node metastasis; LNM‐N2, mediastinal LNM; AUC, area under ROC.

**Figure S2:** Decision curves of the nomograms for predicting LNM (A, B) and LNM‐N2 (C, D) in the development and validation cohorts. The red line represents the nomograms. The gray line represents the assumption that all patients have LNM or LNM‐N2. The black line represents the assumption that no patients have LNM or LNM‐N2. LNM, lymph node metastasis; LNM‐N2, mediastinal LNM.

**Figure S3:** Frequency and location of LNMs in the low‐risk and high‐risk groups stratified according to the LNM nomogram in clinical stage IA lung adenocarcinoma. LNM, lymph node metastasis.

**Figure S4:** Workflow of treatment planning for patients with clinical stage IA lung adenocarcinomas by CT according to the nomograms. CT, computed tomography; pGGO, pure ground‐glass opacity; AAH, atypical adenomatous hyperplasia; AIS, adenocarcinoma in situ; MIA, minimally invasive adenocarcinoma; LNM, lymph node metastasis; LNM‐N2, mediastinal LNM; LML, limited mediastinal lymphadenectomy; SML, systematic mediastinal lymphadenectomy.

## Data Availability

All the data supporting the findings of this study are available from the corresponding author upon reasonable request.
