# Supplementary material for: Nomograms for intraoperative prediction of lymph node metastasis in clinical stage IA lung adenocarcinoma
Source: Cancer Med. 2023 May 22;12(13):14360–74. doi: 10.1002/cam4.6115 (PMC10358245; doi:10.1002/cam4.6115)
Supplement: Supplementary file 1 — Table S1. Univariable and multivariable analyses for recurrence‐free and overall survival [file CAM4-12-14360-s005.docx]

| **Supplementary Table 1.** Univariable and multivariable analyses for recurrence-free and overall survival | | | | | | | | | | | |
| --- | --- | --- | --- | --- | --- | --- | --- | --- | --- | --- | --- |
|  | Recurrence-free survival | | | | |  | Overall survival | | | | |
|  | Univariable Analysis | |  | Multivariable Analysis | |  | Univariable Analysis | |  | Multivariable Analysis | |
| Variables | HR (95% CI) | P value |  | HR (95% CI) | P value |  | HR (95% CI) | P value |  | HR (95% CI) | P value |
| ***Low-risk group for LNM-N2*** | | | | | | | | | | | |
| Age, years |  |  |  |  |  |  |  |  |  |  |  |
| ≦60 | Reference |  |  | Reference |  |  | Reference |  |  | Reference |  |
| >60 | 1.80 (0.99-3.27) | 0.055 |  | 1.01 (0.52-1.96) | 0.983 |  | 2.27 (0.90-5.75) | 0.084 |  | 1.16 (0.40-3.32) | 0.789 |
| Sex |  |  |  |  |  |  |  |  |  |  |  |
| Male | Reference |  |  |  |  |  | Reference |  |  |  |  |
| Female | 0.69 (0.38-1.25) | 0.222 |  |  |  |  | 0.53 (0.21-1.34) | 0.180 |  |  |  |
| Smoking history |  |  |  |  |  |  |  |  |  |  |  |
| Never | Reference |  |  |  |  |  | Reference |  |  | Reference |  |
| Former/Current | 1.53 (0.84-2.80) | 0.163 |  |  |  |  | 2.93 (1.13-7.55) | 0.027 |  | 2.63(0.80-8.64) | 0.112 |
| CEA, ng/mL |  |  |  |  |  |  |  |  |  |  |  |
| <5 | Reference |  |  | Reference |  |  | Reference |  |  | Reference |  |
| ≧5 | 10.46 (4.09-26.75) | <0.001 |  | 4.66 (1.18-18.35) | 0.028 |  | 24.00 (7.75-74.34) | <0.001 |  | 19.18 (3.23-113.84) | 0.001 |
| Tumor location |  |  |  |  |  |  |  |  |  |  |  |
| Upper lobe | Reference |  |  |  |  |  | Reference |  |  |  |  |
| Middle or lower lobe | 0.57 (0.28-1.15) | 0.118 |  |  |  |  | 0.94 (0.35-2.50) | 0.896 |  |  |  |
| CT appearance |  |  |  |  |  |  |  |  |  |  |  |
| Pure-solid | Reference |  |  | Reference |  |  | Reference |  |  |  |  |
| Part-solid | 0.46 (0.25-0.85) | 0.013 |  | 0.99 (0.33-2.97) | 0.982 |  | 0.66 (0.26-1.68) | 0.383 |  |  |  |
| Consolidation/Tumor ratio |  |  |  |  |  |  |  |  |  |  |  |
| ≧50% | Reference |  |  | Reference |  |  | Reference |  |  | Reference |  |
| <50% | 0.23 (0.13-0.42) | <0.001 |  | 0.46 (0.13-1.55) | 0.210 |  | 0.41 (0.16-1.09) | 0.073 |  | 1.23 (0.18-8.43) | 0.833 |
| Pathologic tumor size | 2.06 (1.24-3.42) | 0.005 |  | 0.93 (0.42-2.04) | 0.847 |  | 3.39 (1.62-7.10) | 0.001 |  | 2.52 (0.84-7.56) | 0.099 |
| Pathologic nodal stage |  |  |  |  |  |  |  |  |  |  |  |
| N0 | Reference |  |  | Reference |  |  | Reference |  |  | Reference |  |
| N1 | 4.52 (0.62-32.98) | 0.137 |  | 0.40 (0.03-6.22) | 0.509 |  | - |  |  | - |  |
| N2 | 9.92 (3.53-27.85) | <0.001 |  | 0.45 (0.09-2.20) | 0.325 |  | 26.46 (7.43-94.32) | <0.001 |  | 0.44 (0.06-3.43) | 0.431 |
| Visceral pleural invasion |  |  |  |  |  |  |  |  |  |  |  |
| Absent | Reference |  |  | Reference |  |  | Reference |  |  | Reference |  |
| Present | 6.92 (3.79-12.64) | <0.001 |  | 4.32 (1.81-10.28) | 0.001 |  | 2.51 (0.93-6.82) | 0.070 |  | 2.13 (0.41-11.05) | 0.369 |
| Lymphovascular invasion |  |  |  |  |  |  |  |  |  |  |  |
| Absent | Reference |  |  | Reference |  |  | Reference |  |  | Reference |  |
| Present | 5.55 (2.18-14.13) | <0.001 |  | 0.69 (0.16-2.98) | 0.623 |  | 19.28 (6.81-54.61) | <0.001 |  | 3.35 (0.56-19.85) | 0.183 |
| STAS |  |  |  |  |  |  |  |  |  |  |  |
| Absent | Reference |  |  | Reference |  |  | Reference |  |  | Reference |  |
| Present | 7.34 (3.83-14.07) | <0.001 |  | 6.89 (2.39-19.84) | <0.001 |  | 10.62 (4.10-27.49) | <0.001 |  | 10.97 (2.01-59.79) | 0.006 |
| Micropapillary pattern |  |  |  |  |  |  |  |  |  |  |  |
| Absent | Reference |  |  | Reference |  |  | Reference |  |  | Reference |  |
| Present | 5.05 (2.72-9.38) | <0.001 |  | 2.47 (1.13-5.39) | 0.023 |  | 4.60 (1.78-11.87) | 0.002 |  | 0.75 (0.17-3.29) | 0.705 |
| Solid pattern |  |  |  |  |  |  |  |  |  |  |  |
| Absent | Reference |  |  | Reference |  |  | Reference |  |  | Reference |  |
| Present | 5.54 (2.96-10.38) | <0.001 |  | 1.77 (0.73-4.27) | 0.203 |  | 6.79 (2.68-17.24) | <0.001 |  | 1.40 (0.36-5.40) | 0.622 |
| Lepidic pattern |  |  |  |  |  |  |  |  |  |  |  |
| Absent | Reference |  |  |  |  |  | Reference |  |  |  |  |
| Present | 0.97 (0.49-1.93) | 0.939 |  |  |  |  | 0.50 (0.15-1.74) | 0.278 |  |  |  |
| Lymphadenectomy |  |  |  |  |  |  |  |  |  |  |  |
| SML | Reference |  |  | Reference |  |  | Reference |  |  | Reference |  |
| LML | 0.92 (0.51-1.68) | 0.790 |  | 1.31 (0.65-2.61) | 0.449 |  | 0.66 (0.26-1.67) | 0.377 |  | 2.01 (0.60-6.65) | 0.256 |
| ***High-risk group for LNM-N2*** | | | | | | | | | | | |
| Age, years |  |  |  |  |  |  |  |  |  |  |  |
| ≦60 | Reference |  |  |  |  |  | Reference |  |  |  |  |
| >60 | 1.64 (0.86-3.11) | 0.132 |  |  |  |  | 1.55 (0.69-3.46) | 0.287 |  |  |  |
| Sex |  |  |  |  |  |  |  |  |  |  |  |
| Male | Reference |  |  |  |  |  | Reference |  |  |  |  |
| Female | 0.61 (0.32-1.16) | 0.132 |  |  |  |  | 0.90 (0.40-2.00) | 0.789 |  |  |  |
| Smoking history |  |  |  |  |  |  |  |  |  |  |  |
| Never | Reference |  |  |  |  |  | Reference |  |  |  |  |
| Former/Current | 1.19 (0.63-2.26) | 0.588 |  |  |  |  | 1.46 (0.65-3.24) | 0.358 |  |  |  |
| CEA, ng/mL |  |  |  |  |  |  |  |  |  |  |  |
| <5 | Reference |  |  | Reference |  |  | Reference |  |  | Reference |  |
| ≧5 | 2.34 (1.24-4.43) | 0.009 |  | 1.46 (0.69-3.10) | 0.326 |  | 2.41 (1.08-5.38) | 0.031 |  | 1.69 (0.68 -4.23) | 0.259 |
| Tumor location |  |  |  |  |  |  |  |  |  |  |  |
| Upper lobe | Reference |  |  |  |  |  | Reference |  |  |  |  |
| Middle or lower lobe | 1.05 (0.55-2.01) | 0.893 |  |  |  |  | 1.38 (0.62-3.07) | 0.437 |  |  |  |
| Pathologic tumor size | 1.20 (0.53-2.69) | 0.663 |  |  |  |  | 1.25 (0.47-3.32) | 0.650 |  |  |  |
| Pathologic nodal stage |  |  |  |  |  |  |  |  |  |  |  |
| N0 | Reference |  |  | Reference |  |  | Reference |  |  | Reference |  |
| N1 | 1.03 (0.24-4.36) | 0.971 |  | 1.10 (0.24-4.95) | 0.903 |  | 0.90 (0.12-6.84) | 0.921 |  | 1.90 (0.24-15.31) | 0.547 |
| N2 | 2.81 (1.40-5.63) | 0.004 |  | 2.50 (1.06-5.91) | 0.037 |  | 3.08 (1.30-7.28) | 0.011 |  | 1.94 (0.71-5.28) | 0.196 |
| Visceral pleural invasion |  |  |  |  |  |  |  |  |  |  |  |
| Absent | Reference |  |  | Reference |  |  | Reference |  |  |  |  |
| Present | 1.98 (0.94-4.19) | 0.073 |  | 1.14 (0.48-2.72) | 0.762 |  | 0.84 (0.25-2.81) | 0.773 |  |  |  |
| Lymphovascular invasion |  |  |  |  |  |  |  |  |  |  |  |
| Absent | Reference |  |  | Reference |  |  | Reference |  |  |  |  |
| Present | 3.28 (1.66-6.52) | 0.001 |  | 1.90 (0.84-4.26) | 0.122 |  | 1.77 (0.66-4.74) | 0.258 |  |  |  |
| STAS |  |  |  |  |  |  |  |  |  |  |  |
| Absent | Reference |  |  | Reference |  |  | Reference |  |  |  |  |
| Present | 1.74 (0.92-3.28) | 0.090 |  | 2.36 (1.06-5.22) | 0.035 |  | 1.92 (0.86-4.28) | 0.112 |  |  |  |
| Micropapillary pattern |  |  |  |  |  |  |  |  |  |  |  |
| Absent | Reference |  |  |  |  |  | Reference |  |  |  |  |
| Present | 1.31 (0.69-2.49) | 0.400 |  |  |  |  | 0.98 (0.43-2.25) | 0.962 |  |  |  |
| Solid pattern |  |  |  |  |  |  |  |  |  |  |  |
| Absent | Reference |  |  |  |  |  | Reference |  |  | Reference |  |
| Present | 1.59 (0.84-3.02) | 0.152 |  |  |  |  | 4.20 (1.74-10.15) | 0.001 |  | 3.96 (1.54-10.21) | 0.004 |
| Lepidic pattern |  |  |  |  |  |  |  |  |  |  |  |
| Absent | Reference |  |  |  |  |  | Reference |  |  |  |  |
| Present | 1.00 (0.31-3.26) | 0.995 |  |  |  |  | 0.50 (0.07-3.71) | 0.499 |  |  |  |
| Lymphadenectomy |  |  |  |  |  |  |  |  |  |  |  |
| SML | Reference |  |  | Reference |  |  | Reference |  |  | Reference |  |
| LML | 1.95 (1.03-3.69) | 0.040 |  | 4.00 (1.82-8.75) | 0.001 |  | 2.28 (1.02-5.09) | 0.044 |  | 3.30 (1.44-7.57) | 0.005 |

LNM, lymph node metastasis; LNM-N2, mediastinal LNM; HR, hazard ratio; CI, confidence interval; CEA, serum carcinoembryonic antigen; CT, computed tomography; STAS, tumor spread through air spaces; SML, systematic mediastinal lymphadenectomy; LML, limited mediastinal lymphadenectomy.
