# Supplementary material for: Nomograms for intraoperative prediction of lymph node metastasis in clinical stage IA lung adenocarcinoma
Source: Cancer Med. 2023 May 22;12(13):14360–74. doi: 10.1002/cam4.6115 (PMC10358245; doi:10.1002/cam4.6115)
Supplement: Supplementary file 2 — Table S2. Baseline characteristics of patients undergoing systematic or limited mediastinal lymphadenectomy [file CAM4-12-14360-s001.docx]

| **Supplementary Table 2.** Baseline characteristics of patients undergoing systematic or limited mediastinal lymphadenectomy | | | | | | | | | | | |  |
| --- | --- | --- | --- | --- | --- | --- | --- | --- | --- | --- | --- | --- |
|  | Low-risk group for LNM-N2 | | | |  | | High-risk group for LNM-N2 | | | | |  |
| Characteristics | SML (n=204) |  | LML (n=219) | P | |  | | SML (n=104) |  | LML (n=59) | P | |
| Age, years |  |  |  |  | |  | |  |  |  |  | |
| ≦60 | 131 (64.2) |  | 147 (67.1) | 0.529 | |  | | 61 (58.6) |  | 26 (44.1) | 0.073 | |
| >60 | 73 (35.8) |  | 72 (32.9) |  | |  | | 43 (41.4) |  | 33 (55.9) |  | |
| Sex |  |  |  |  | |  | |  |  |  |  | |
| Male | 80 (39.2) |  | 77 (35.2) | 0.388 | |  | | 41 (39.4) |  | 28 (47.5) | 0.318 | |
| Female | 124 (60.8) |  | 142 (64.8) |  | |  | | 63 (60.6) |  | 31 (52.5) |  | |
| Smoking history |  |  |  |  | |  | |  |  |  |  | |
| Never | 131 (64.2) |  | 147 (67.1) | 0.529 | |  | | 59 (56.7) |  | 36 (61.0) | 0.594 | |
| Former/Current | 73 (35.8) |  | 72 (32.9) |  | |  | | 45 (43.3) |  | 23 (39.0) |  | |
| CEA, ng/mL |  |  |  |  | |  | |  |  |  |  | |
| ≧5 | 5 (2.5) |  | 3 (1.4) | 0.415 | |  | | 70 (67.3) |  | 46 (78.0) | 0.149 | |
| <5 | 199 (97.5) |  | 216 (98.6) |  | |  | | 34 (32.7) |  | 13 (22.0) |  | |
| Tumor location |  |  |  |  | |  | |  |  |  |  | |
| Right upper lobe | 74 (36.3) |  | 76 (34.7) | 0.016 | |  | | 26 (25.0) |  | 14 (23.7) | 0.334 | |
| Right middle lobe | 15 (7.3) |  | 26 (11.9) |  | |  | | 7 (6.7) |  | 5 (8.5) |  | |
| Right lower lobe | 48 (23.5) |  | 29 (13.2) |  | |  | | 28 (26.9) |  | 12 (20.4) |  | |
| Left upper lobe | 33 (16.2) |  | 54 (24.7) |  | |  | | 32 (30.8) |  | 15 (25.4) |  | |
| Left lower lobe | 34 (16.7) |  | 34 (15.5) |  | |  | | 11 (10.6) |  | 13 (22.0) |  | |
| CT appearance |  |  |  |  | |  | |  |  |  |  | |
| Part-solid | 89 (43.6) |  | 90 (41.1) | 0.598 | |  | | 0 |  | 0 | - | |
| Pure-solid | 115 (56.4) |  | 129 (58.9) |  | |  | | 104 (100.0) |  | 59 (100.0) |  | |
| Consolidation/Tumor ratio |  |  |  |  | |  | |  |  |  |  | |
| ≧50% | 39 (19.1) |  | 33 (15.1) | 0.268 | |  | | 104 (100.0) |  | 59 (100.0) | - | |
| <50% | 165 (80.9) |  | 186 (84.9) |  | |  | | 0 |  | 0 |  | |
| Pathologic tumor size, cm | 1.34 ± 0.03 |  | 1.28± 0.03 | 0.219 | |  | | 2.12 ± 0.04 |  | 2.04± 0.04 | 0.222 | |
| Pathologic nodal stage |  |  |  |  | |  | |  |  |  |  | |
| N0 | 197 (96.5) |  | 218 (99.5) | 0.069 | |  | | 72 (69.2) |  | 54 (91.53) | 0.005 | |
| N1 | 3 (1.5) |  | 0 (0.0) |  | |  | | 9 (8.7) |  | 1 (1.7) |  | |
| N2 | 4 (2.0) |  | 1 (0.5) |  | |  | | 23 (22.1) |  | 4 (6.8) |  | |
| Visceral pleural invasion |  |  |  |  | |  | |  |  |  |  | |
| Absent | 180 (88.2) |  | 190 (86.8) | 0.647 | |  | | 90 (86.5) |  | 51 (86.4) | 0.986 | |
| Present | 24 (11.8) |  | 29 (13.2) |  | |  | | 14 (13.5) |  | 8 (13.6) |  | |
| Lymphovascular invasion |  |  |  |  | |  | |  |  |  |  | |
| Absent | 199 (97.5) |  | 212 (96.8) | 0.645 | |  | | 89 (85.6) |  | 51 (86.4) | 0.879 | |
| Present | 5 (2.5) |  | 7 (3.2) |  | |  | | 15 (14.4) |  | 8 (13.6) |  | |
| STAS |  |  |  |  | |  | |  |  |  |  | |
| Absent | 184 (90.2) |  | 213 (97.3) | 0.003 | |  | | 49 (47.1) |  | 51 (86.4) | <0.001 | |
| Present | 20 (9.8) |  | 6 (2.7) |  | |  | | 55 (52.9) |  | 8 (13.6) |  | |
| Micropapillary pattern |  |  |  |  | |  | |  |  |  |  | |
| Absent | 183 (89.7) |  | 189 (86.3) | 0.283 | |  | | 57 (54.8) |  | 38 (64.4) | 0.232 | |
| Present | 21 (10.3) |  | 30 (13.7) |  | |  | | 47 (45.2) |  | 21 (35.6) |  | |
| Solid pattern |  |  |  |  | |  | |  |  |  |  | |
| Absent | 178 (87.3) |  | 204 (93.1) | 0.041 | |  | | 59 (56.7) |  | 41 (69.5) | 0.108 | |
| Present | 26 (12.7) |  | 15 (6.9) |  | |  | | 45 (43.3) |  | 18 (30.5) |  | |
| Lepidic pattern |  |  |  |  | |  | |  |  |  |  | |
| Absent | 161 (78.9) |  | 156 (71.2) | 0.068 | |  | | 96 (92.3) |  | 55 (93.2) | 0.830 | |
| Present | 43 (21.1) |  | 63 (28.8) |  | |  | | 8 (7.7) |  | 4 (6.8) |  | |

LNM, lymph node metastasis; LNM-N2, mediastinal LNM; SML, systematic mediastinal lymphadenectomy; LML, limited mediastinal lymphadenectomy; CEA, serum carcinoembryonic antigen; CT, computed tomography; STAS, tumor spread through air spaces.
