# Supplementary figures and images for: Nomograms for intraoperative prediction of lymph node metastasis in clinical stage IA lung adenocarcinoma
Source: Cancer Med. 2023 May 22;12(13):14360–74. doi: 10.1002/cam4.6115 (PMC10358245; doi:10.1002/cam4.6115)

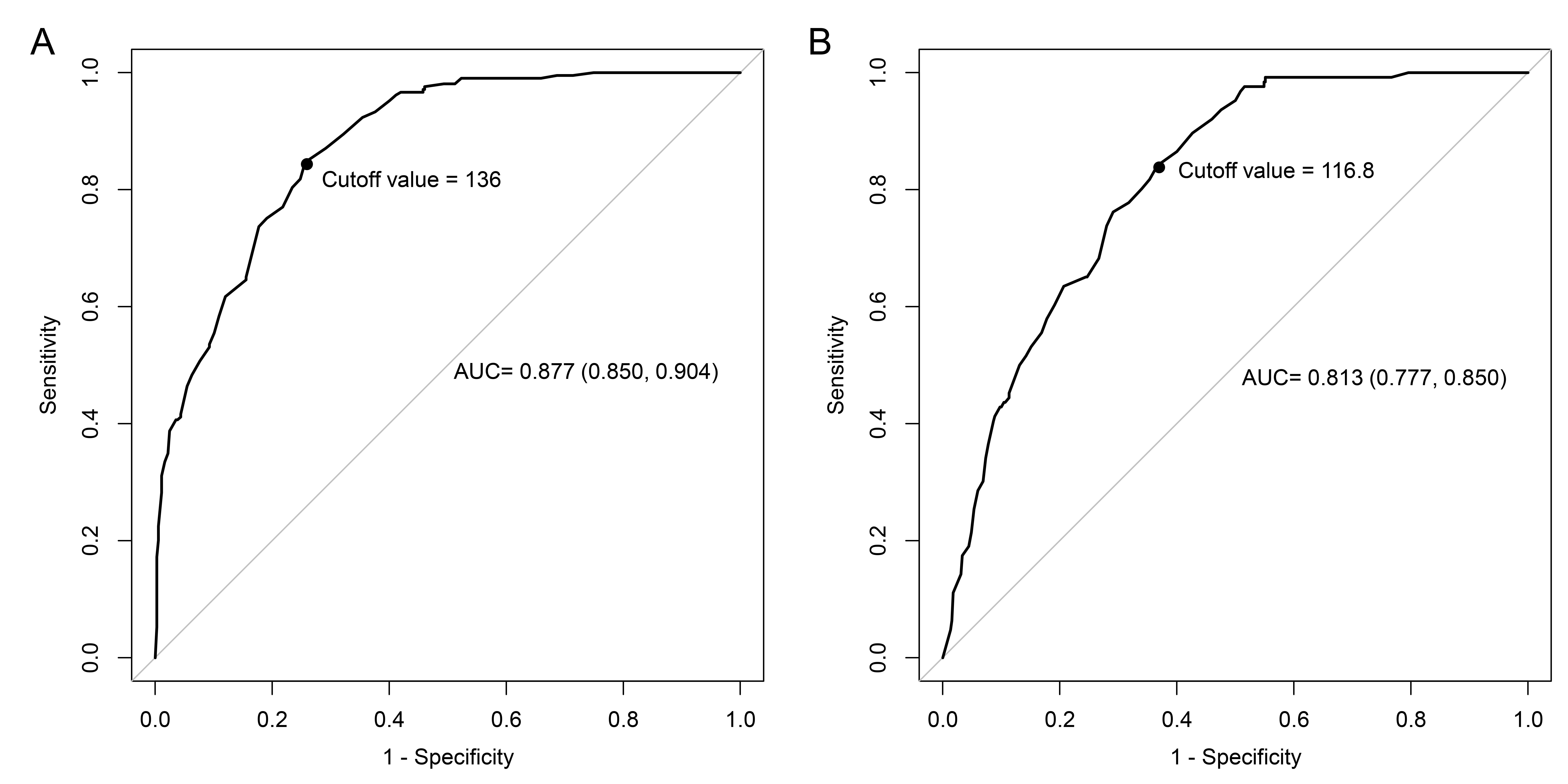

Supplement: Supplementary file 3 — Figure S1: ROC curves of the nomograms for predicting LNM (A) and LNM‐N2 (B) according to the optimal cutoff points determined by the maximum Youden Index. ROC, receiver operating characteristic; LNM, lymph node metastasis; LNM‐N2, mediastinal LNM; AUC, area under ROC. [file CAM4-12-14360-s003.tif]

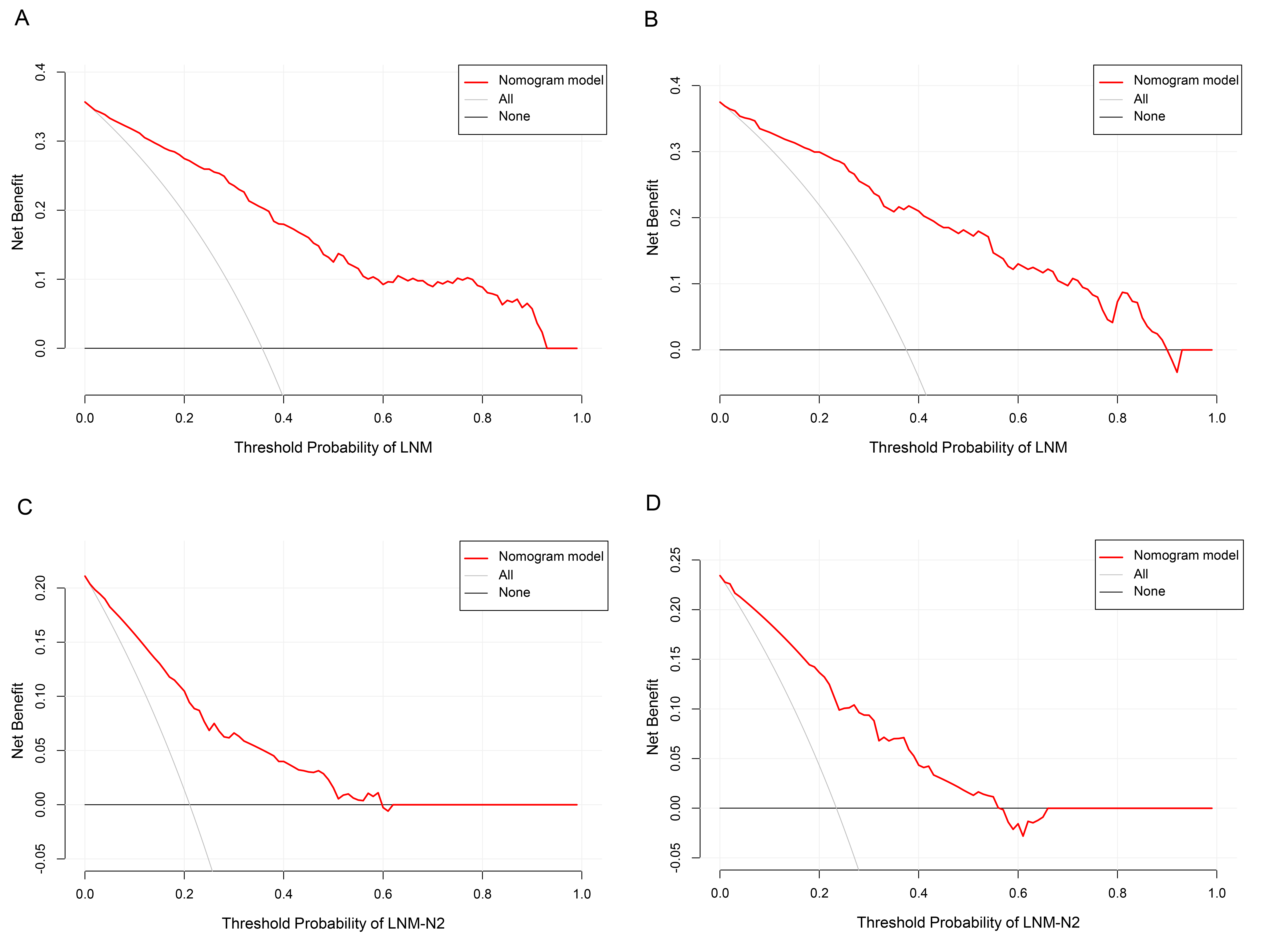

Supplement: Supplementary file 4 — Figure S2: Decision curves of the nomograms for predicting LNM (A, B) and LNM‐N2 (C, D) in the development and validation cohorts. The red line represents the nomograms. The gray line represents the assumption that all patients have LNM or LNM‐N2. The black line represents the assumption that no patients have LNM or LNM‐N2. LNM, lymph node metastasis; LNM‐N2, mediastinal LNM. [file CAM4-12-14360-s004.tif]

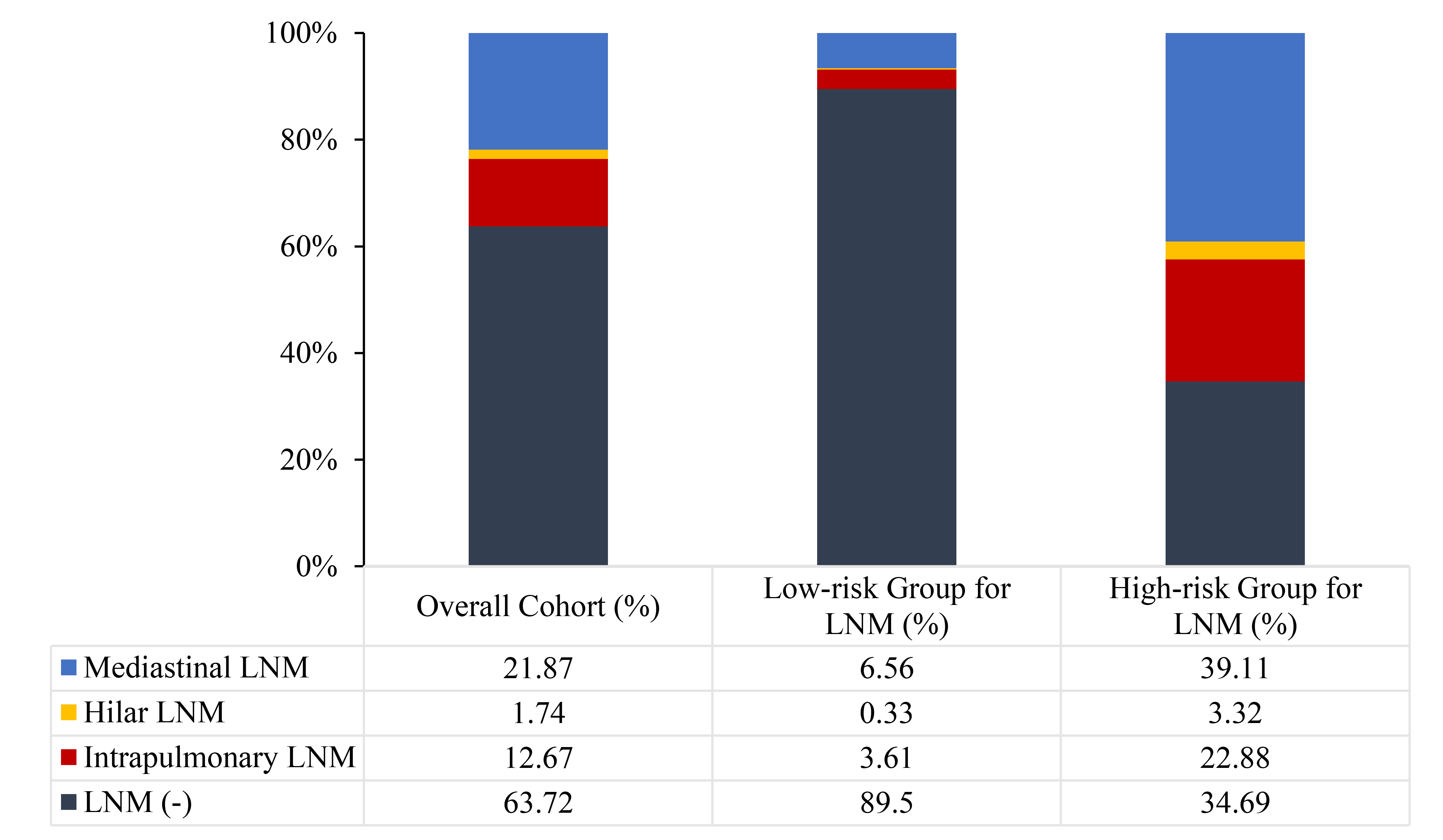

Supplement: Supplementary file 5 — Figure S3: Frequency and location of LNMs in the low‐risk and high‐risk groups stratified according to the LNM nomogram in clinical stage IA lung adenocarcinoma. LNM, lymph node metastasis. [file CAM4-12-14360-s002.tif]

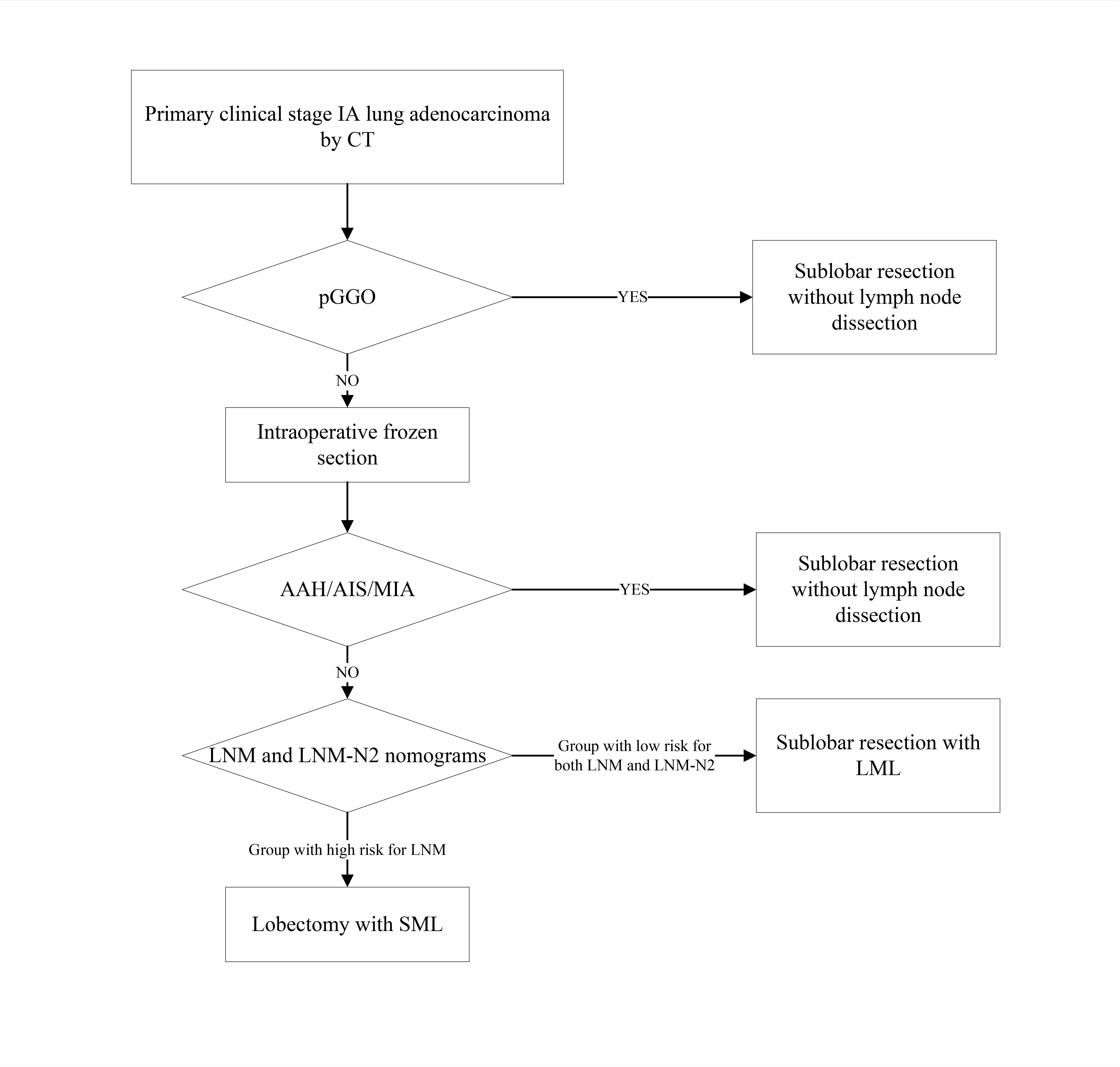

Supplement: Supplementary file 6 — Figure S4: Workflow of treatment planning for patients with clinical stage IA lung adenocarcinomas by CT according to the nomograms. CT, computed tomography; pGGO, pure ground‐glass opacity; AAH, atypical adenomatous hyperplasia; AIS, adenocarcinoma in situ; MIA, minimally invasive adenocarcinoma; LNM, lymph node metastasis; LNM‐N2, mediastinal LNM; LML, limited mediastinal lymphadenectomy; SML, systematic mediastinal lymphadenectomy. [file CAM4-12-14360-s006.tif]
